# Association between Diurnal Variation of Ozone Concentration and Stroke Occurrence: 24-Hour Time Series Study

**DOI:** 10.1371/journal.pone.0152433

**Published:** 2016-03-25

**Authors:** Myung-Hoon Han, Hyeong-Joong Yi, Young-Seo Kim, Yong Ko, Young-Soo Kim

**Affiliations:** 1 Department of Neurosurgery, Hanyang University Medical Center, 222–1, Wangsimni-ro, Seongdong-gu, Seoul, Korea; 2 Department of Neurology, Hanyang University Medical Center, 222–1, Wangsimni-ro, Seongdong-gu, Seoul, Korea; 3 Department of Neurosurgery, Hanyang University Guri Hospital, 153 Gyeongchun-ro, Guri 471–701, Gyonggi-do, Korea; National Health Research Institutes, TAIWAN

## Abstract

**Background and Purpose:**

Increasing ozone concentrations have been known to damage human health and ecosystems. Although ozone tends to display diurnal variation, most studies have reported only on the association between daily ozone concentrations and ischemic stroke occurrence on the same day, or with a 1-day lag. We investigated the effect of the diurnal variation of ozone on ischemic stroke occurrence during the same day.

**Methods:**

We included a consecutive series of 1,734 patients from January 1, 2008, to December 31, 2014, at a single tertiary hospital in Seoul, South Korea. We evaluated differences between temperature and pollutants at the time of stroke onset for each time interval and averaged those parameters across the 7-year study period.

**Results:**

During the interval from 13:00 to 16:59, we found a positive association between ischemic stroke occurrence and ozone concentration relative to other time periods. Upper median ozone levels from 13:00 to 16:59 were positively correlated with ischemic stroke (odds ratio, 1.550; 95% confidence intervals, 1.220 to 1.970; *P* = <0.001) when compared with lower median levels.

**Conclusions:**

The results show diurnal patterns of ischemic stroke occurrence based on upper and lower median ozone levels for a 24-hour period, which extends understanding of the association between stroke occurrence and environmental influences.

## Introduction

Every year in Korea, approximately 105,000 people experience new or recurrent stroke, and more than 26,000 people die of stroke. In other words, every 5 minutes, someone in Korea experiences a stroke, and every 20 minutes, a stroke-related death occurs.[1] Many epidemiological studies have linked meteorological factors or concentrations of air pollutants with ischemic stroke (IS).[1–9] Ozone (O_3_) is the air pollutant most consistently projected to increase as a result of future climate change.[10] The increasing tropospheric concentrations of O_3_ have received extensive attention around the world because of the related damage to human health and ecosystems.[11–14] However, inconsistent and incomplete information has been reported on the relationship between IS occurrence and O_3_ concentrations. Some studies[15–21] have asserted that IS occurrence is associated with O_3_, whereas other studies[22–26] have reported no association between them. However, none of those studies investigated the relationship between IS occurrence and O_3_ levels using time-specific IS onset and diurnal variation of O_3_ concentrations over 24-h periods. We hypothesized that diurnal variations of ozone concentration could affect ischemic stroke occurrence. In addition, because studies[2,17,27–29] have reported an association between IS occurrence and temperature and particulate matter less than 10 μm in aerodynamic diameter (PM_10_), we also evaluated the effects of those factors on IS occurrence across 24-h periods.

## Materials and Methods

### Study area

The Seongdong district is in an urban section of Seoul in northern South Korea with a mean population of about 250,000. Hanyang University Medical Center is the sole regional tertiary hospital qualified to treat stroke in the Seongdong district. We previously reported the characteristics of the Seongdong district.[30] Patients within the study area can reach the Hanyang University Medical Center emergency unit within 15 minutes by car, and almost all emergency patients within the Seongdong district are transported to our hospital according to the guidelines of the Emergency Medical Services system.

### Stroke registry and patients

**Fig 1:**
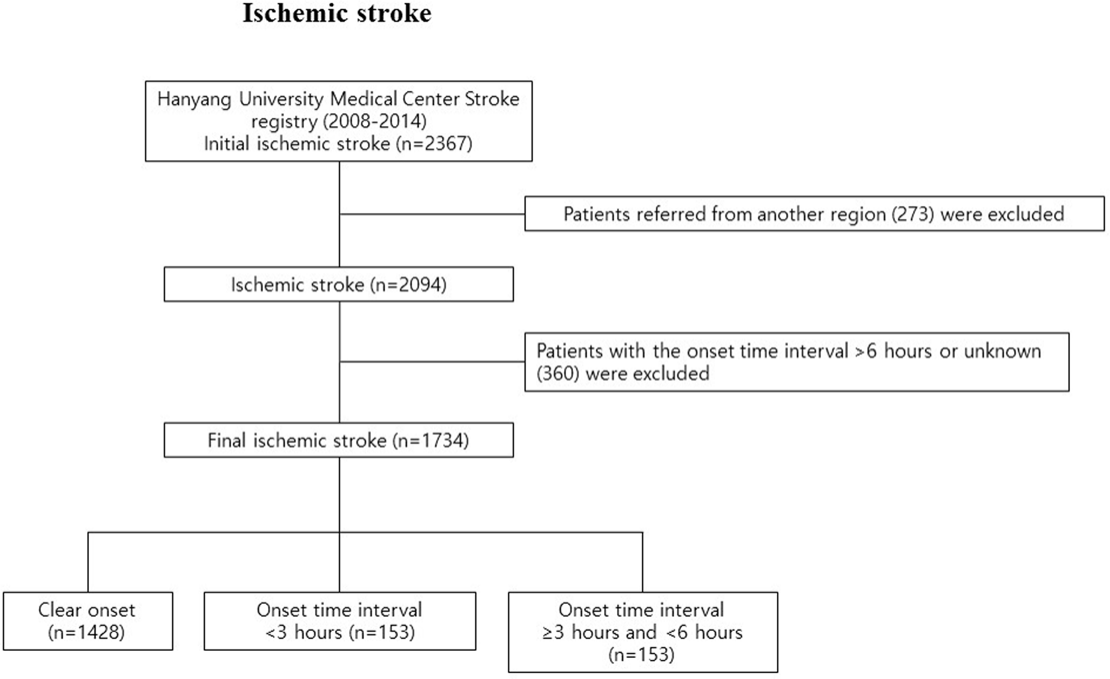
Flow chart of the process for selecting eligible patients for our study from the Hanyang University Medical Center Stroke Registry during the period from January 1, 2008, to December 31, 2014.

We collected patient data retrospectively from the Hanyang University Medical Center Stroke Registry. In the end, we included a consecutive series of 1,734 patients from January 1, 2008, to December 31, 2014 (Fig 1).

We included recurrent stroke patients because a previous study reported no differences in circadian variation in recurrent stroke compared with first stroke.[31]

The Hanyang University Medical Center Stroke Registry (established in 2007) was designed for prospective research, and the registration system is well organized. The data quality, consistency, and accuracy of our registry are reliable because trained staff manage all data directly and consistently within a single hospital for various research purposes. A neurologist diagnosed IS based on clinical symptoms following the World Health Organization criteria,[32] as well as neurologic imaging using CT or MRI, in all cases. This study was approved by the institutional review board of Hanyang University Medical Center. Due to the retrospective nature of this study, the ethics committee did not require subsequent informed written consent of the included patients. However, we de-identified and anonymized patient records prior to analysis.

### Analysis of onset time

The time (registered per minute) and situation at IS onset are recorded in detail in our registry. When patients perceived the symptoms of stroke or guardians observed the occurrence of stroke in patients, we used the time of perception or observation as the onset time (clear onset). However, when the precise onset time was not clear (unclear onset), mostly due to sleep, we investigated and recorded in our registration system the last time that a patient felt or was seen to be normal and the first time a patient felt or was seen to be abnormal. We excluded patients with time intervals of more than six hours between the last normal time and first abnormal time because we expected high levels of heterogeneity in the time of IS occurrence, air temperature, and pollutant concentrations when we redistributed those patients across more than six hours. In addition, we were afraid of losing too many patients to overnight sleep if we reduced the range of time intervals too far to enhance the reliability. We therefore divided unclear onset patients within 6 hours into 0:01 to 2:59 hours and 3:00 to 5:59 hours and redistributed them to the middle of the preceding pertinent time intervals.

### Temperature and air pollution variables

We investigated hourly temperature data in the Seongdong district for the day of stroke occurrence in all cases for the 7-year study period. Then we registered the temperature at the time of stroke onset. In addition, we collected hourly mean temperatures for every month from January 2008 to December 2014 (S1 Table). We obtained those data from the Meteorological Administration of South Korea (http://www.kma.go.kr). Similarly, we collected data on pollutants, including measures of PM_10_ and O_3_ in the Seongdong district, Seoul, for the 7-year study period from the Climate and Air Quality Management Division of South Korea (http://www.airkorea.or.kr).

### Statistical methods

We present baseline characteristics of patient data as mean ± standard deviation and number/percentage. We included patients with both clear and unclear onset (time interval 0–6h) in all analyses. We used the Chi-square test and one-way ANOVA to assess differences in the variables at 4-hour intervals.

We used descriptive statistics to describe the hourly incidence of IS and hourly measures of temperature, PM_10_, and O_3_ at the time of stroke onset from January 1, 2008, to December 31, 2014.

We categorized temperature, PM10 and O3 variables for the time of day of stroke onset into quartile groups and lower and upper median groups based on average hourly temperature, PM10 and O3 for every month during the study period for each 4-hour time interval, to evaluate possible differences between temperature, PM10 and O3 of the time of day of stroke onset for each time interval and averaged those parameters for each time interval during the study period. Then we calculated the number of IS occurrences based on the quartile and lower and upper median groups for temperature, PM_10_, and O_3_ stratified by 4-hour intervals. In addition, we divided the O_3_ variable for the time of stroke onset into lower and upper median groups based on the average hourly O_3_ for every month during the study period at 1-hour intervals.

We estimated the odds ratio (OR) with 95% confidence intervals (CIs) using multinomial logistic regression of IS occurrence for each 4-hour interval based on the lower and upper median groups for temperature, PM_10_, and O_3_ to evaluate the association of those variables with IS occurrence (S3 Table). We divided temperature, PM_10_, and O_3_ at the time of stroke onset (in 4-hour intervals) into lower and upper median groups for the same intervals using the average hourly values for every month during the study period (S2 Table). Then we calculated the ORs of the lower (coding = 1) and upper (coding = 2) median groups for temperature, PM_10_, and O_3_ for each 4-hour interval compared with half of the total number of IS occurrences (coding = 0) for each interval.

We also divided the day into 4-hour intervals to identify potential differences in IS occurrence when comparing the temperature, PM_10_, and O_3_ in each 4-hour interval with those in other time periods. Assuming that IS onset is not related to temperature, PM_10_, or O_3_ for each 4-hour interval, we calculated the OR with 95% CIs, using uni- and multivariable binary logistic regression of IS incidence for each 4-hour interval. First, we categorized temperature, PM_10_, and O_3_ at the time of stroke occurrence into the lower (coding = 0) or upper median group (coding = 1) as covariates for each 4-hour interval, based on the median and average hourly values for every month of the study period. Second, we coded 1 for the interval we wanted to calculate as a dependent variable and coded 0 for the other intervals. We first calculated the OR of IS occurrence using a univariate logistic regression model for each 4-hour time interval alternatively with covariates of median group variables (reference = lower median group) for temperature, PM_10_, and O_3_. We then estimated ORs with 95% CIs using a multivariate logistic regression model to adjust for possible confounders and considering *p*<0.05 as statistically significant.

All statistical analyses were performed using SPSS for Windows, version 22.0.

## Results

We included 1,734 patients with IS; 373 patients (21.5%) in the 09:00–13:00-h interval and 355 patients (20.5%) in the 13:00–17:00-h interval. The average age of IS onset was 66.29 years, and 57.4% of patients were men. There were no significant differences between the time intervals and the prevalence of stroke risk factors except for the history of alcohol drinking (*P* = 0.022). Further descriptive data stratified by 4-hour intervals, including TOAST classification, history of risk factors, and recurrent stroke, are shown in Table 1.

**Table 1:** Characteristics of Patients with Ischemic Stroke in the Hanyang University Medical Center Stroke Registry, and Distribution of Variables Based on 4-hour Time Intervals from January 1, 2008, to December 31, 2014.

		Time intervals						
Variables	All patients	01:00–04:59	05:00–08:59	09:00–12:59	13:00–16:59	17:00–20:59	21:00–00:59	*P*
Number (%)	1734	113 (6.5)	345 (19.9)	373 (21.5)	355 (20.5)	301 (17.4)	247 (14.2)	
Sex, male, n (%)	996 (57.4)	63 (55.8)	210 (60.9)	223 (59.8)	193 (54.4)	171 (56.8)	136 (55.1)	0.464*
Age, mean (SD), y	66.29 (13.06)	64.47 (13.72)	65.49 (13.74)	67.87 (12.25)	66.00 (13.39)	66.69 (13.04)	65.77 (12.35)	0.416†
Age group								
<40, n (%)	60 (3.5)	6 (5.3)	15 (4.3)	10 (2.7)	16 (4.5)	6 (2.0)	7 (2.8)	0.302*
40–59, n (%)	446 (25.7)	32 (28.3)	89 (25.8)	81 (21.7)	102 (28.7)	74 (24.6)	68 (27.5)	0.322*
≥60, n (%)	1228 (70.8)	75 (66.4)	241 (69.9)	282 (75.6)	237 (66.8)	221 (73.4)	172 (69.6)	0.096*
TOAST subtype								
LAA, n (%)	517 (29.8)	22 (19.5)	129 (37.4)	110 (29.5)	102 (28.7)	82 (27.2)	72 (29.1)	0.006*
SAO, n (%)	560 (32.3)	36 (31.9)	116 (33.6)	130 (34.9)	112 (31.5)	93 (30.9)	73 (29.6)	0.760*
CE, n (%)	374 (21.6)	28 (24.8)	63 (18.3)	70 (18.8)	81 (22.8)	73 (24.3)	59 (23.9)	0.217*
Other, n (%)	66 (3.8)	6 (5.3)	4 (1.2)	18 (4.8)	13 (3.7)	16 (5.3)	9 (3.6)	0.068*
Undetermined, n (%)	217 (12.5)	21 (18.6)	33 (9.6)	45 (12.1)	47 (13.2)	37 (12.3)	34 (13.8)	0.210*
Risk factors								
Hypertension, n (%)	948 (54.7)	63 (55.8)	165 (47.8)	220 (59.0)	197 (55.5)	173 (57.5)	130 (52.6)	0.055*
Diabetes mellitus, n (%)	511 (29.5)	30 (26.5)	90 (26.1)	119 (31.9)	116 (32.7)	89 (29.6)	67 (27.1)	0.322*
Smoking, n (%)	387 (22.3)	22 (19.5)	75 (21.7)	86 (23.1)	67 (18.9)	76 (25.2)	61 (24.7)	0.365*
Alcohol drinking, n (%)	621 (35.8)	34 (30.1)	107 (31.0)	138 (37.0)	133 (37.5)	101 (33.6)	108 (43.7)	0.022*
Hyperlipidemia, n (%)	321 (18.5)	20 (17.7)	78 (22.6)	60 (16.1)	68 (19.2)	45 (15.0)	50 (20.2)	0.133*
Atrial fibrillation, n (%)	168 (9.7)	10 (8.8)	45 (13.0)	30 (8.0)	30 (8.5)	31 (10.3)	22 (8.9)	0.252*
Recurrent stroke, n (%)	271 (15.6)	14 (12.4)	50 (14.5)	61 (16.4)	53 (14.9)	50 (16.6)	43 (17.4)	0.802*

*Chi-square test

†ANOVA test

SD, standard deviation; LAA, large artery atherosclerosis; SVO, small vessel occlusion; CE, cardioembolism

Table 2 shows the number of IS patients with clear onset times within 6-hour intervals and the temperature, PM_10_, and O_3_ at the time of stroke occurrence stratified by 1-hour intervals. In addition, we also present the median and quartiles 1 and 3 of hourly average temperature, PM_10_, and O_3_ for every month during the 7-year study period stratified by 1-hour and 4-hour intervals as a reference (S1 and S2 Tables).

**Table 2:** Descriptive Analysis of the Number of Stroke Patients and Hourly Temperature and Air Pollutants on Day of Stroke Occurrence from January 1, 2008, to December 31, 2014, in the Seongdong district, Seoul, Korea.

	Clear onset	Time interval 0–6 h	Temperature (°C)			PM_10_ (μg/m^3^)			O_3_ (ppb)		
Time	Number	Number	Mean	Min	Max	Mean	Min	Max	Mean	Min	Max
01:00–01:59	10	17	11.01	-13.60	29.00	46.52	28.00	84.00	14.22	7.00	29.00
02:00–02:59	4	21	9.57	-13.60	24.90	45.64	26.00	73.00	16.08	7.00	27.00
03:00–03:59	14	16	9.76	-12.70	27.40	45.47	28.00	72.00	16.97	9.00	29.00
04:00-04-59	13	18	9.91	-6.10	25.20	42.55	26.00	62.00	15.87	8.00	25.00
05:00–05:59	8	16	13.04	-9.40	24.80	40.33	30.00	56.00	15.79	9.00	24.00
06:00–06:59	34	18	10.06	-12.40	26.00	40.96	20.00	68.00	13.88	6.00	51.00
07:00–07:59	108	19	8.25	-13.40	26.70	45.26	20.00	74.00	10.66	4.00	20.00
08:00–08:59	118	24	10.92	-15.00	26.30	44.23	23.00	75.00	9.80	4.00	19.00
09:00–09:59	102	11	10.71	-12.70	28.60	46.11	24.00	76.00	12.51	4.00	23.00
10:00–10:59	72	16	13.52	-11.30	29.70	46.55	7.00	83.00	15.72	6.00	29.00
11:00–11:59	62	9	11.96	-6.90	29.20	49.73	24.00	92.00	20.35	8.00	45.00
12:00–12:59	87	14	18.12	-8.70	33.30	44.75	21.00	100.00	26.92	10.00	45.00
13:00–13:59	64	5	17.45	-7.60	33.00	46.75	25.00	76.00	34.83	16.00	54.00
14:00–14:59	87	3	16.15	-9.60	32.80	47.22	20.00	100.00	36.18	15.00	62.00
15:00–15:59	80	14	18.71	-7.50	35.10	43.10	21.00	75.00	36.49	15.00	68.00
16:00–16:59	90	12	14.59	-12.50	33.70	49.38	21.00	85.00	37.27	14.00	70.00
17:00–17:59	74	5	13.85	-11.60	33.70	49.15	21.00	78.00	35.78	9.00	63.00
18:00–18:59	68	10	15.31	-8.30	30.80	47.10	23.00	80.00	28.67	8.00	57.00
19:00–19:59	66	5	13.21	-9.30	30.60	49.76	23.00	81.00	25.04	6.00	48.00
20:00–20:59	62	11	14.18	-6.00	28.20	50.56	3.00	86.00	20.49	6.00	40.00
21:00–21:59	52	8	11.20	-7.80	28.70	54.80	28.00	94.00	16.40	6.00	34.00
22:00–22:59	54	3	14.08	-8.10	27.20	52.70	29.00	100.00	15.70	6.00	27.00
23:00–23:59	29	11	10.21	-11.60	27.40	53.43	30.00	100.00	14.53	7.00	47.00
00:00–00:59	70	20	10.24	-11.30	26.10	50.84	28.00	108.00	13.33	6.00	25.00

PM_10_, particulate matter less than 10 μm in aerodynamic diameter; O_3_, ozone; Min, minimum; Max, maximum

**Fig 2:**
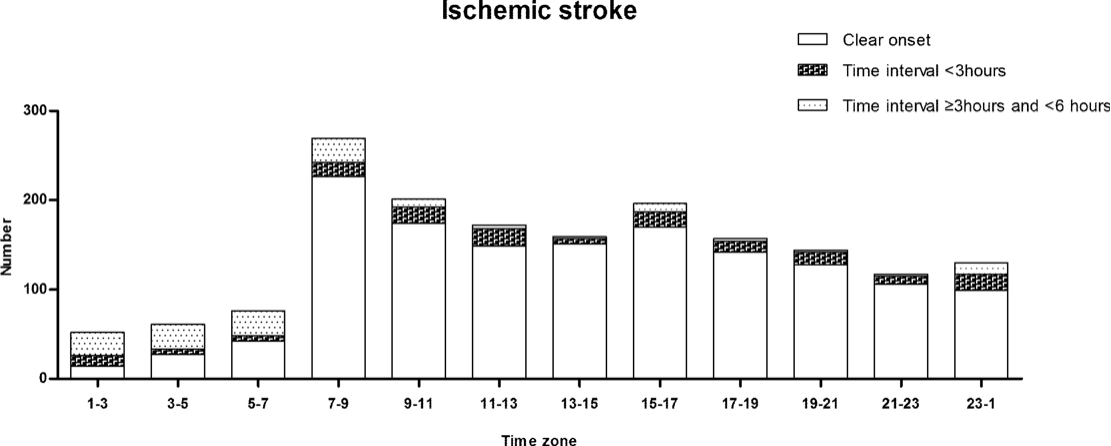
Distribution of time-specific IS with clear onset and unclear onset ranges from 0–3 h and 3–6 h, all assigned within 2-hour intervals.

Fig 2 shows the number of IS with clear onset and unclear onset ranges from 0–3 h and 3–6 h, all assigned within 2-hour intervals.

IS occurrence peaked during the period from 07:00 to 08:59, with 226 patients with clear onset, 16 with unclear onset within 3 hours and 27 with unclear onset between 3 and 6 hours.

**Fig 3:**
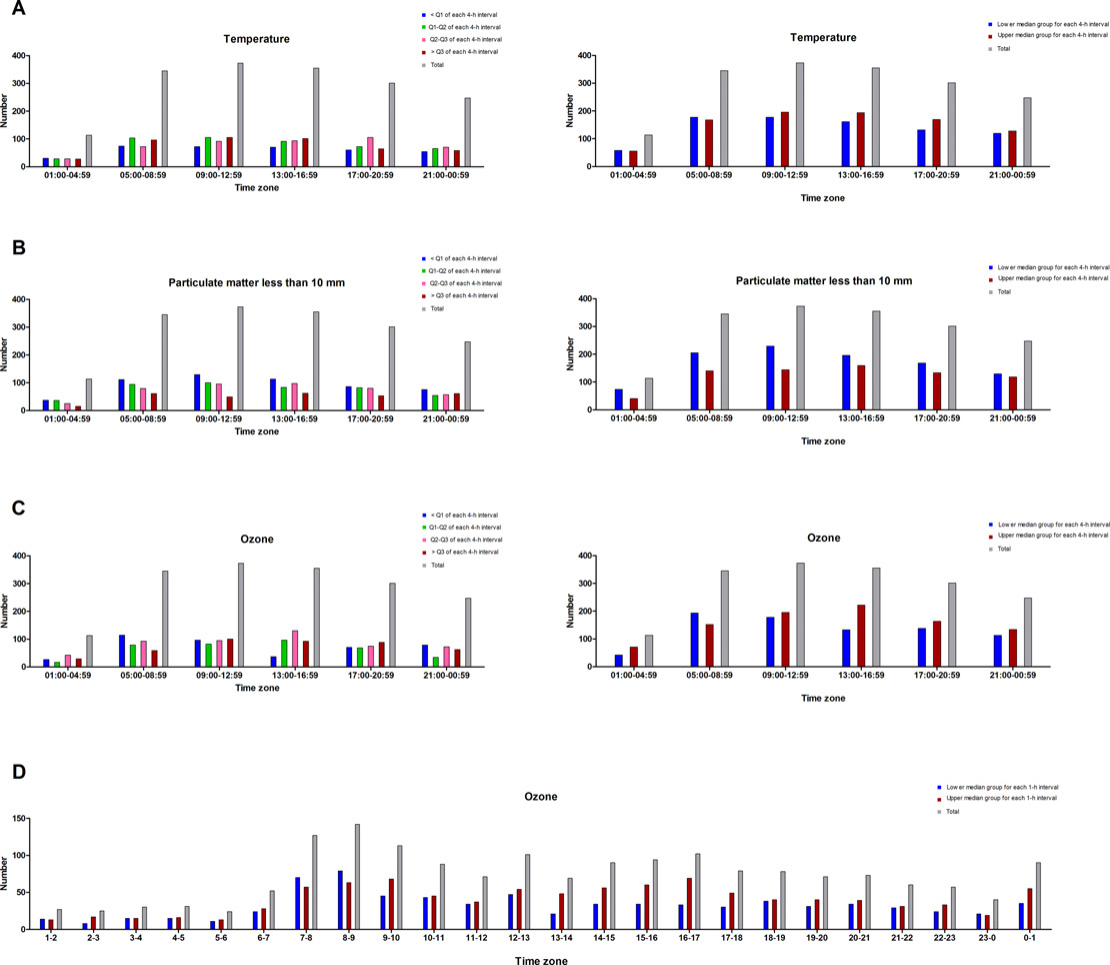
Time-specific stroke onset pattern with data on temperature, PM10, and O3 for the time of stroke onset divided into quartiles and medians for each 4-hour interval and O3 for the time of stroke onset divided into medians for each 1-hour interval by the average hourly values for every month during the 7-year study period.

Fig 3 presents time-specific IS onset patterns with data on temperature, PM_10_, and O_3_ for the time of stroke onset divided into quartiles and medians for each 4-hour interval and O_3_ for the time of stroke onset divided into medians for each 1-hour interval by the average hourly values for every month during the 7-year study period.

The upper median groups of temperature for each 4-hour interval show patterns of slightly higher IS occurrence than the lower median groups, whereas a tendency toward higher IS occurrence appears in the lower median group for PM_10_. We found a higher IS occurrence in the upper median group of O_3_ compared with the lower median group during the period from 13:00 to 16:59. In addition, we observed a higher IS occurrence in the upper median group from 13:00 to 17:59 in the 1-hour interval model for O_3_.

We estimated ORs with 95% CIs for IS occurrence using a multinomial logistic regression model. We found a positive association between IS occurrence and the upper median group of O_3_ (OR, 1.255; 95% CI, 1.021 to 1.510; *P* = 0.017) relative to half of the total number of IS onsets from 13:00 to 16:59 (S3 Table).

Table 3 shows ORs with 95% CIs for IS occurrence stratified by 4-hour intervals based on temperature, PM_10_, and O_3_. Each 4-hour interval contains a similar number of patients. There was a negative relationship between IS occurrence and O_3_ concentration during the period from 05:00 to 08:59 (OR, 0.635; 95% CI, 0.492 to 0.819; P<0.001) after full adjustment. In the period from 13:00 to 16:59, we found a positive association between IS occurrence and O_3_ concentration relative to the other time periods. The upper median group of O_3_ from 13:00 to 16:59 was positively correlated with IS (OR, 1.550; 95% CI, 1.220 to 1.970; *P*<0.001) compared with the lower median group of O_3_. These data suggest that the upper median group of O_3_ is associated with an approximately 1.5-fold higher risk of IS occurrence relative to the lower median group from 13:00 to 16:59 compared with the other time periods. In addition, this positive association between higher O_3_ concentrations and IS occurrence from 13:00 to 16:59 was maintained after full adjustment for PM_10_, temperature, sex, age, and risk factors (OR, 1.515; 95% CI, 1.172 to 1.959; *P* = 0.002). A higher IS occurrence was observed in the higher ozone concentration only in the top-tier ozone concentration during the period from 13:00 to 17:00 compared to the other time periods. In the other intervals, IS occurrence was not significantly higher in the upper median group for ozone compared to the lower median group. In this study, we observed about a 1.5-fold higher risk of IS occurrence at an ozone concentration above 33 ppb than below that threshold. Thus, IS might be associated with ozone concentrations above that level.

**Table 3:** Odds Ratios and 95% Confidence Intervals for Each 4-Hour Interval of Ischemic Stroke Occurrence Based on the Upper Median Group Compared with the Lower Median Group of Temperature, PM_10_, and O_3_ for the Hour the Stroke Occurred Categorized by the Median of Average Hourly Values for Every Month During the 7-Year Study Period.

			Time interval (hour)			
	01:00–04:59	05:00–08:59	09:00–12:59	13:00–16:59	17:00–20:59	21:00–00:59
	(n = 113)	(n = 345)	(n = 373)	(n = 355)	(n = 301)	(n = 247)
	OR (95% CI)	OR (95% CI)	OR (95% CI)	OR (95% CI)	OR (95% CI)	OR (95% CI)
Unadjusted						
Temperature (°C)	0.820 (0.560–1.201)	0.830 (0.656–1.051)	1.006 (0.800–1.266)	1.119 (0.885–1.414)	1.199 (0.934–1.540)	0.975 (0.765–1.243)
PM_10_ (μg/m3)	0.732 (0.492–1.090)	0.914 (0.719–1.162)	0.822 (0.650–1.039)	1.134 (0.897–1.435)	1.096 (0.853–1.408)	1.170 (0.916–1.493)
O_3_ (ppb)	1.181 (0.803–1.736)	0.617 (0.486–0.782)†	0.930 (0.740–1.170)	1.550 (1.220–1.970)†	1.023 (0.797–1.312)	0.885 (0.694–1.129)
Model 1						
PM_10_ (μg/m^3^)	0.724 (0.486–1.079)	0.940 (0.738–1.196)	0.825 (0.652–1.043)	1.106 (0.873–1.400)	1.095 (0.852–1.407)	1.180 (0.923–1.507)
O_3_ (ppb)	1.203 (0.817–1.770)	0.619 (0.488–0.785)†	0.941 (0.747–1.184)	1.541 (1.212–1.959)†	1.017 (0.792–1.306)	0.876 (0.687–1.118)
Model 2						
Temperature (°C)	0.619 (0.401–0.954)*	0.903 (0.691–1.179)	0.937 (0.719–1.220)	1.041 (0.795–1.362)	1.328 (0.993–1.777)	1.107 (0.836–1.467)
PM_10_ (μg/m^3^)	0.598 (0.388–0.922)*	0.903 (0.691–1.179)	0.803 (0.620–1.040)	1.123 (0.866–1.457)	1.231 (0.931–1.627)	1.230 (0.938–1.612)
O_3_ (ppb)	1.356 (0.902–2.041)	0.638 (0.495–0.821)†	0.960 (0.752–1.225)	1.505 (1.166–1.942)*	0.929 (0.711–1.213)	0.848 (0.654–1.100)
Model 3						
Temperature (°C)	0.633 (0.410–0.977)*	0.921 (0.698–1.214)	0.940 (0.721–1.226)	1.052 (0.802–1.380)	1.294 (0.965–1.737)	1.086 (0.818–1.442)
PM_10_ (μg/m^3^)	0.602 (0.390–0.928)*	0.912 (0.697–1.193)	0.797 (0.615–1.033)	1.105 (0.851–1.436)	1.232 (0.929–1.635)	1.235 (0.940–1.623)
O_3_ (ppb)	1.342 (0.890–2.024)	0.635 (0.492–0.819)†	0.957 (0.749–1.223)	1.515 (1.172–1.959)*	0.939 (0.717–1.230)	0.846 (0.651–1.099)

Model 1 was adjusted for each pollutant (PM_10_ and O_3_); Model 2 was adjusted for the variables in Model 1 plus temperature; Model 3 was adjusted for the variables in Model 2 plus age, sex and history of risk factors. PM_10_, particulate matter less than 10 mm in aerodynamic diameter; O_3_, ozone; OR, Odds ratio

*p<0.05

†*p*<0.001, respectively

## Discussion

The present study shows IS onset patterns for each time interval according to quartile and lower and upper median groups of temperature and air pollution concentrations. Our results accord with those of previous studies that reported higher rates of IS occurrence in the morning.[31,33–35] We also observed an abrupt decrease in IS occurrence from 10:00 to 11:59 and a gradual slight increase in IS onset from early afternoon to late afternoon. We found a significant association between higher IS occurrence and upper median O_3_ concentrations between 13:00 and 16:59. We observed that the effect of O_3_ remained significant during that time, compared with the other time periods, after including temperature, PM_10_, and other risk factors in the logistic model. In addition, there was a tendency toward higher IS occurrence in the higher O_3_ group in the afternoon, which increased gradually from 13:00–13:59 and peaked from 16:00–16:59. We also found a tendency for higher IS occurrence in the lower median groups of PM_10_. However, we found no significant association between IS occurrence and PM_10_ concentrations. These findings are in line with a recent meta-analysis[36] that reported that PM_10_ showed non-significant associations with hospital admission for IS.

O_3_ shows definite diurnal variation. According to the Climate and Air Quality Management Division of South Korea, O_3_ concentration begins to increase after sunrise, 08:00–09:00 h, and attains its maximum level in the afternoon at 14:00–15:00 h. During the late evening hours, 18:00–20:00 h, the surface O_3_ concentration tends to decrease and reaches a minimum. High temperatures, solar radiation, and longer sunshine duration lead to higher concentrations of O_3_.[37] Therefore, we think it valuable to evaluate the immediate relationship between stroke occurrence and O_3_ concentrations based on 24-hour time series data.

First, we need to clarify the units of measure used for O_3_ because previous studies used several O_3_ units. Using a conversion program, we converted O_3_ units between ppm, ppb, and μg/m^3^. One ppb is 1/1000 ppm, and assuming an ambient pressure of 1 atmosphere and a temperature of 25 degrees, 1 ppb O_3_ is approximately 2.00 μg/m^3^.

Many studies have reported an association between IS occurrence and O_3_ concentration,[15–21] and most studies have described short-term effects (0–2 day lag) for O_3_ on IS occurrence.[16,19–21] On the other hand, some studies found that IS occurrence was not associated with O_3_.[22–26] However, all of those studies investigated the relationship between IS occurrence or mortality and O_3_ using daily O_3_ values (average, 8-hour, or 1-hour maximum or interquartile range increase). As explained above, we think that our 24-hour time series study is more meaningful for an evaluation of the direct and immediate effect of O_3_ on IS occurrence because O_3_ concentrations vary dramatically from morning to afternoon. Although we found a slight increase in IS occurrence during the high O_3_ period in our study, we found higher proportions of high O_3_ concentration groups than low O_3_ groups among the total IS occurrences in the high O_3_ period. In addition, the higher O_3_ groups in the high O_3_ period showed a significant association with higher IS occurrence relative to the lower O_3_ groups when compared with the other time periods. In our study, IS occurrence showed a gradual increase in the group with higher levels of O_3_ from around 13:00 and reached a peak during the period from 16:00–16:59. This finding suggests that IS occurrence might increase depending on exposure time to higher O_3_ concentrations during periods of high levels of O_3._

We classified O_3_ at the time of stroke onset from 13:00 to 16:59 by the median (33 ppb) of the average hourly O_3_ for every month during the study period during the same time intervals. However, 33 ppb of O_3_ was the median of O_3_ from 13:00 to 16:59 for the study period. Therefore, it is unclear whether O_3_ above 33 ppb during a high O_3_ period really affects IS occurrence. A study in the USA[14] reported that the ambient concentration–response relationship for O_3_ showed evidence of a threshold at a little over 30 ppb. In other words, adverse health effects exist above that threshold. Similar findings were reported by a more recent study in Korea and Japan.[13] Those authors reported that the range of Japanese and Korean city thresholds for a link between the daily mean ambient O_3_ concentration and the daily number of non-accidental deaths was from 11 to 34 ppb. The same city-combined analysis also showed a non-linear association with a threshold of 30–40 ppb with a 0–1 day lag. In addition, Ren et al.[12] reported short-term effects for O_3_ on stroke mortality. They found that exposure to O_3_ was strongly associated with mortality in diabetes and stroke, associated a little more strongly with respiratory disorders, and weakly associated with heart disease, such as myocardial infarction or cardiovascular diseases. Our study also showed higher IS occurrence in the upper median group (>33ppb of ozone) in the top-tiered ozone concentration during the period from 13:00 to 17:00. Therefore, we hypothesize that an O_3_ concentration of just over 30 ppb might have some effect on stroke occurrence.

Some pathophysiological hypotheses could explain our findings. Devlin et al.[38] demonstrated O_3_-induced changes in several markers associated with fibrinolysis in response to fibrin deposition in 23 healthy young volunteers. They reported that a small increase in tissue-type plasminogen activator was seen immediately after a 2-hour exposure to ozone. Although O_3_ concentrations in the high O_3_ period in our study were not high as the O_3_ exposure (0.3 ppm) over 2 hours in that study, the subjects in that study were exposed to high levels of ozone for only 2 hours, in contrast to real-life situations that would involve much lengthier ozone exposures. A study in Canada[39] reported that short-term inhalation of fine particulate (PM_2.5_) air pollution and ozone at concentrations that occur in urban environments caused acute conduit artery vasoconstriction. These vasoconstrictor effects might induce obstruction in a stenotic artery, giving rise to ischemia, or trigger the rupture of an unstable atherosclerosis plaque. The mostly older patients in our study with vascular disease might be predisposed to thromboembolic episodes. Dales et al.[40] found a significant association between exposure to O_3_ and venous thrombosis in a study in the Netherlands,[41] which reported that exposure to air pollutants such as O_3_ was associated with increased platelet aggregation and thrombin generation.

We observed higher IS occurrence in lower O_3_ concentrations during the period from 05:00 to 09:00. Ozone forms through the splitting of nitrogen dioxide by sunlight to provide the primary source of the oxygen atoms required for O_3_ formation.[42] Sunlight splits nitrogen dioxide into nitric oxide and an oxygen atom (NO_2_ + sunlight → NO + O). A single oxygen atom then combines with an oxygen molecule to produce O_3_ (O_2_ + O → O_3_). Therefore, there is an inverse diurnal relationship between O_3_ and nitrogen dioxide.[43] The study also showed higher concentrations of both carbon monoxide and PM_2.5_ during the period from 05:00 to 09:00. A recent meta-analysis[25] reported that PM_2.5_, nitrogen dioxide, and carbon monoxide were associated with the risk of hospital admission for stroke. However, PM_10_ was not significantly related to the risk of stroke admission. We think higher concentrations of PM_2.5_, nitrogen dioxide, and carbon monoxide, which are inversely related to ozone, might be significantly associated with higher IS occurrence between 05:00 and 09:00.

Generally, IS occurrence tended to rise slightly in the high temperature group (upper median group). Tsai et al.[17] reported that O_3_ showed a significant association with IS occurrence on warm days. In addition, we found no positive association between IS onset and higher PM_10_ concentrations. Xiang et al.[44] explained that the main reason for higher PM_10_ levels in cold temperatures are air stagnation caused by light wind, lack of precipitation, and formation of an inversion layer. These conditions make it hard for air-suspended particles to diffuse to higher altitudes. Therefore, we assume that PM_10_ levels might have been relatively low on the relatively warm days on which stroke occurred in our study. A recent meta-analysis[45] reported that PM_2.5_ and PM_10_ were associated with higher total cerebrovascular disease mortality, whereas those pollutants showed inconsistent, non-significant associations with hospital admission for IS.

Our study has some limitations. First, it is from only one region, and the generalizability of our findings is therefore limited. However, population characteristics, including exposure levels and socioeconomic factors, are likely to be more homogenous within small geographical areas.[46] Therefore, studies covering a larger region have inevitable data inconsistency issues, as well as a lack of weather and environmental homogeneity. Shah et al.[25] reported that many studies measured concentrations of air pollution at remote monitoring sites, and therefore some degree of misclassification of exposure is likely in those studies. Second, personal exposure to pollutants might differ from pollutant levels at fixed outdoor monitoring stations. Older people might have disabilities or limited mobility and thus could spend most of their time indoors. According to the Ministry of Health and Welfare in Korea, as lifespan has increased with medical advances in Korea, older people in Korea have shown greater interest in activities such as golf, mountain climbing, light jogging, and other outdoor activities. Some studies have reported that O_3_ levels were positively correlated with IS occurrence at a 0- or 1-day lag in the elderly.[16,19,20] The third limitation is the possibility of stroke onset time error. However, most unclear-onset patients redistributed to the time between 13:00 and 16:59 had less than 3 hours of time between their last certain normal time and their first abnormal time. Therefore we think this error was unlikely to have skewed the main findings, though it might have decreased the statistical power. Finally, we could not evaluate the lag-day effect of O_3_ levels on IS occurrence. However, most studies[16,19–21] have found that O_3_ was associated with IS occurrence with a 0- to 1-day lag. Therefore our study would show the immediate O_3_ effect on IS occurrence with a 0-day lag.

In conclusion, we demonstrated IS onset patterns for each time interval based on hourly temperature and air pollution concentration data for the day of stroke occurrence, and we classified those values using their averages across the study period. Our results suggest that exposure to O_3_ during periods of high O_3_ concentration, such as from 13:00 to 18:00, might be associated with higher IS occurrence. Our study of 24-hour time series data to evaluate the effect of diurnal variation of O_3_ on IS occurrence extends understanding of the association between stroke occurrence and environmental influences.

## Supporting Information

S1 Table Descriptive statistics for hourly average temperature, PM10, and O3 for every month from January 2008 to December 2014 in Seongdong district, Seoul, Korea. (DOCX)

S2 Table Descriptive statistics for 4-hour time intervals of average temperature, PM10, and O3 for every month from January 2008 to December 2014 in Seongdong district, Seoul, Korea. (DOCX)

S3 Table Odds ratios and 95% confidence intervals for ischemic stroke occurrence based on temperature, PM10, and O3 at the hour of stroke occurrence categorized by median groups of average hourly temperature, PM10, and O3 for every month of the 7-year study period. (DOCX)
