# Supplementary material for: Association between Diurnal Variation of Ozone Concentration and Stroke Occurrence: 24-Hour Time Series Study
Source: PLoS One. 2016 Mar 25;11(3):e0152433. doi: 10.1371/journal.pone.0152433 (PMC4807846; doi:10.1371/journal.pone.0152433)
Supplement: S1 Table — (DOCX) [file pone.0152433.s001.docx]

|  |  |  | Temperature (°C) | | | |  | PM_10_ (μg/m^3^) | | | |  | O_3_ (ppb) | | | |
| --- | --- | --- | --- | --- | --- | --- | --- | --- | --- | --- | --- | --- | --- | --- | --- | --- |
| Time |  | n | Mean | Min | Median | Max |  | Mean | Min | Median | Max |  | Mean | Min | Median | Max |
| 1-2 |  | 84 | 10.72 | -10.50 | 11.45 | 27.00 |  | 49.89 | 21.00 | 49.50 | 86.00 |  | 13.52 | 6.00 | 12.00 | 25.00 |
| 2-3 |  | 84 | 10.40 | -10.90 | 10.95 | 26.80 |  | 48.43 | 21.00 | 48.00 | 81.00 |  | 14.85 | 7.00 | 13.00 | 27.00 |
| 3-4 |  | 84 | 10.11 | -11.10 | 10.60 | 26.40 |  | 47.17 | 21.00 | 46.50 | 74.00 |  | 15.68 | 7.00 | 14.00 | 29.00 |
| 4-5 |  | 84 | 9.78 | -11.40 | 10.25 | 25.90 |  | 46.06 | 20.00 | 46.00 | 74.00 |  | 15.93 | 8.00 | 14.00 | 29.00 |
| 5-6 |  | 84 | 9.59 | -11.80 | 10.50 | 26.00 |  | 45.46 | 20.00 | 46.00 | 74.00 |  | 15.32 | 7.00 | 14.00 | 27.00 |
| 6-7 |  | 84 | 9.43 | -12.20 | 10.60 | 26.20 |  | 45.49 | 20.00 | 46.50 | 73.00 |  | 13.10 | 6.00 | 12.00 | 24.00 |
| 7-8 |  | 84 | 9.50 | -12.40 | 10.50 | 26.50 |  | 45.49 | 20.00 | 45.00 | 74.00 |  | 10.51 | 4.00 | 10.00 | 20.00 |
| 8-9 |  | 84 | 10.10 | -12.60 | 11.45 | 27.00 |  | 47.12 | 20.00 | 47.50 | 75.00 |  | 9.94 | 4.00 | 9.00 | 21.00 |
| 9-10 |  | 84 | 11.10 | -12.30 | 13.30 | 27.80 |  | 49.00 | 20.00 | 49.50 | 79.00 |  | 11.77 | 4.00 | 10.50 | 23.00 |
| 10-11 |  | 84 | 12.29 | -11.30 | 14.30 | 29.00 |  | 50.48 | 21.00 | 51.00 | 83.00 |  | 15.32 | 6.00 | 13.00 | 29.00 |
| 11-12 |  | 84 | 13.39 | -10.60 | 15.90 | 30.40 |  | 51.02 | 21.00 | 51.50 | 92.00 |  | 19.81 | 8.00 | 18.00 | 36.00 |
| 12-13 |  | 84 | 14.22 | -10.30 | 16.75 | 30.90 |  | 50.43 | 21.00 | 51.00 | 100.00 |  | 24.81 | 10.00 | 24.00 | 45.00 |
| 13-14 |  | 84 | 14.87 | -10.60 | 17.90 | 31.70 |  | 48.82 | 21.00 | 48.00 | 101.00 |  | 30.11 | 13.00 | 29.00 | 54.00 |
| 14-15 |  | 84 | 15.21 | -10.90 | 18.40 | 32.10 |  | 48.83 | 20.00 | 47.00 | 100.00 |  | 34.04 | 15.00 | 33.00 | 62.00 |
| 15-16 |  | 84 | 15.25 | -11.30 | 17.65 | 32.50 |  | 49.25 | 21.00 | 47.00 | 95.00 |  | 35.57 | 15.00 | 34.50 | 68.00 |
| 16-17 |  | 84 | 15.11 | -12.50 | 16.80 | 32.70 |  | 49.49 | 21.00 | 48.50 | 85.00 |  | 35.27 | 14.00 | 34.00 | 70.00 |
| 17-18 |  | 84 | 14.63 | -13.50 | 16.85 | 32.40 |  | 50.23 | 21.00 | 48.50 | 78.00 |  | 31.82 | 12.00 | 30.00 | 67.00 |
| 18-19 |  | 84 | 13.75 | -14.00 | 15.65 | 31.80 |  | 51.13 | 22.00 | 49.50 | 80.00 |  | 27.69 | 8.00 | 28.00 | 59.00 |
| 19-20 |  | 84 | 12.85 | -14.50 | 14.30 | 30.10 |  | 51.24 | 23.00 | 49.50 | 81.00 |  | 24.04 | 6.00 | 23.00 | 48.00 |
| 20-21 |  | 84 | 12.08 | -14.80 | 13.30 | 29.10 |  | 51.20 | 24.00 | 50.00 | 86.00 |  | 19.12 | 6.00 | 17.00 | 40.00 |
| 21-22 |  | 84 | 11.54 | -15.10 | 13.05 | 28.70 |  | 52.98 | 26.00 | 52.00 | 94.00 |  | 16.20 | 6.00 | 14.00 | 33.00 |
| 22-23 |  | 84 | 11.06 | -15.20 | 12.85 | 28.40 |  | 54.19 | 25.00 | 54.50 | 100.00 |  | 14.63 | 6.00 | 13.00 | 29.00 |
| 23-0 |  | 84 | 10.68 | -15.50 | 12.45 | 28.20 |  | 52.46 | 25.00 | 53.50 | 100.00 |  | 13.48 | 6.00 | 12.00 | 25.00 |
| 0-1 |  | 84 | 10.31 | -15.70 | 11.00 | 28.00 |  | 51.15 | 23.00 | 51.00 | 108.00 |  | 12.89 | 6.00 | 11.00 | 25.00 |

PM_10_, particulate matter less than 10 mm in aerodynamic diameter; O_3_, ozone; Min, minimum; Max, maximum
