# Supplementary material for: Association between Diurnal Variation of Ozone Concentration and Stroke Occurrence: 24-Hour Time Series Study
Source: PLoS One. 2016 Mar 25;11(3):e0152433. doi: 10.1371/journal.pone.0152433 (PMC4807846; doi:10.1371/journal.pone.0152433)
Supplement: S2 Table — (DOCX) [file pone.0152433.s002.docx]

|  | Mean temperature (°C) | | | PM_10_ (μg/m^3^) | | | O_3_ (ppb) | | |
| --- | --- | --- | --- | --- | --- | --- | --- | --- | --- |
| Time period | Mean (SD) | Median | Q1, Q3 | Mean (SD) | Median | Q1, Q3 | Mean (SD) | Median | Q1, Q3 |
| 01:00-04:59 | 10.25 | 10.95 | 0.50, 20.48 | 47.89 | 47.00 | 38.25, 56.00 | 14.99 | 13.00 | 11.00, 19.75 |
| 05:00-08:59 | 9.65 | 10.60 | -0.30, 20.25 | 45.89 | 46.00 | 37.00, 57.00 | 12.22 | 11.50 | 8.00, 16.00 |
| 09:00-12:59 | 12.75 | 14.25 | 2.23, 23.45 | 50.23 | 51.00 | 39.00, 62.00 | 17.93 | 16.00 | 11.00, 23.00 |
| 13:00-16:59 | 15.11 | 18.00 | 5.25, 25.50 | 49.10 | 47.50 | 38.00, 61.00 | 33.74 | 33.00 | 22.00, 43.00 |
| 17:00-20:59 | 13.33 | 13.80 | 2.90, 24.18 | 50.95 | 49.00 | 42.00, 62.00 | 25.67 | 26.00 | 15.00, 34.00 |
| 21:00-00:59 | 10.90 | 11.75 | 0.90, 21.80 | 52.70 | 52.00 | 43.00, 61.00 | 14.30 | 12.00 | 9.00, 19.00 |

PM_10_, particulate matter less than 10 mm in aerodynamic diameter; O_3_, ozone; SD, standard deviation; Q, quartile
