# Supplementary material for: Association between Diurnal Variation of Ozone Concentration and Stroke Occurrence: 24-Hour Time Series Study
Source: PLoS One. 2016 Mar 25;11(3):e0152433. doi: 10.1371/journal.pone.0152433 (PMC4807846; doi:10.1371/journal.pone.0152433)
Supplement: S3 Table — (DOCX) [file pone.0152433.s003.docx]

|  |  |  | Time period (hour) |  |  |  |
| --- | --- | --- | --- | --- | --- | --- |
|  | 01:00-04:59  (n=113) | 05:00-08:59  (n=345) | 09:00-12:59  (n=373) | 13:00-16:59  (n=355) | 17:00-20:59  (n=301) | 21:00-00:59  (n=247) |
|  | OR (95% CI) | OR (95% CI) | OR (95% CI) | OR (95% CI) | OR (95% CI) | OR (95% CI) |
| Temperature (°C) |  |  |  |  |  |  |
|  |  |  |  |  |  |  |
| Lower median group | 1.053 (0.728-1.455) | 1.029 (0.854-1.278) | 0.951 (0.771-1.178) | 0.909 (0.727-1.132) | 0.880 (0.678-1.113) | 1.016 (0.791-1.315) |
| Upper median group | 0.965 (0.646-1.405) | 0.976 (0.790-1.215) | 1.053 (0.860-1.284) | 1.096 (0.904-1.346) | 1.126 (0.886-1.402) | 0.992 (0.783-1.285) |
|  |  |  |  |  |  |  |
| PM_10_ (μg/m^3^) |  |  |  |  |  |  |
|  |  |  |  |  |  |  |
| Lower median group | 1.303 (0.909-1.868) | 1.192 (0.984-1.467) | 1.231 (1.020-1.507)* | 1.107 (0.918-1.357) | 1.120 (0.895-1.401) | 1.114 (0.871-1.409) |
| Upper median group | 0.715 (0.467-1.044) | 0.814 (0.652-1.019) | 0.774 (0.619-0.960)* | 0.899 (0.726-1.106) | 0.887 (0.703-1.137) | 0.894 (0.681-1.154) |
|  |  |  |  |  |  |  |
| O_3_ (ppb) |  |  |  |  |  |  |
|  |  |  |  |  |  |  |
| Lower median group | 0.857 (0.586-1.275) | 1.122 (0.912-1.369) | 0.957 (0.770-1.161) | 0.751 (0.605-0.943)* | 0.887 (0.675-1.115) | 0.910 (0.706-1.191) |
| Upper median group | 1.161 (0.797-1.728) | 0.883 (0.712-1.087) | 1.048 (0.875-1.275) | 1.255 (1.021-1.510)* | 1.120 (0.886-1.409) | 1.097 (0.864-1.439) |

PM_10_, particulate matter less than 10 mm in aerodynamic diameter; O_3_, ozone; OR, Odds ratio

**p*<0.05
